# Prostatosymphyseal Fistula After Photoselective Vaporization of the Prostate: A Very Rare Complication of a Transurethral Surgery

**DOI:** 10.7759/cureus.7703

**Published:** 2020-04-16

**Authors:** Filipos Kapogiannis, Konstantinos Fasoulakis, Eleni Tsiampa, Spyridon Triantafyllou, Charalampos Fasoulakis

**Affiliations:** 1 Urology, Hippokration General Hospital, Athens, GRC; 2 Obstetrics and Gynecology, Elena Venizelou General Hospital, Athens, GRC; 3 Urology, Mediterraneo Hospital, Athens, GRC; 4 Urology, National and Kapodistrian University of Athens Medical School, Athens, GRC

**Keywords:** prostatosymphyseal fistula, greenlight laser, radical prostatectomy, photoselective vaporization

## Abstract

Prostatosymphyseal fistula (PSF) is a very rare complication described after transurethral surgery of the prostate including photoselective vaporization of the prostate (PVP) with GreenLight^ΤΜ^ laser (Boston Scientific, Marlborough, MA). Sporadic cases have also been reported in the literature as side effects of pelvic radiation therapy. We present a 65-year-old male patient who underwent PVP as an elective procedure for the treatment of severe lower urinary tract symptoms. The primary management after the diagnosis of the fistula was conservative but as this did not result in an expected improvement, the patient underwent radical prostatectomy as a last resort. PSF formation is the result of the communication between the anterior capsule of the prostate and the bladder neck via pubic symphysis and the surrounding tissues. This condition often leads to either urinoma formation or osteitis pubis. In the majority of cases, treatment options are complex surgical reconstruction using flaps or grafts, radical prostatectomy or urinary diversion as an ultimate solution. The rarity of the complication and the accompanied atypical signs and symptoms warrant a low threshold for suspicion so as to diagnose the event early and provide the appropriate treatment.

## Introduction

Photoselective vaporization of the prostate (PVP) or GreenLight^TM^ laser (Boston Scientific, Marlborough, MA) has become an important tool of the modern endourology for the treatment of lower urinary tract symptoms (LUTS) due to benign prostatic hyperplasia (BPH). Prostatosymphyseal fistula (PSF) formation is a rare complication and has been anticipated practically for any form of transurethral prostate surgery for BPH that expends energy to the prostatic tissue. PSFs occur when the anatomic integrity of the lower urinary tract system is compromised, allowing urine leakage into the surrounding tissues and thus causing a wide spectrum of signs and symptoms from pelvic pain to recurrent urinary tract infections. A literature search found less than 10 reported cases which were treated predominantly with surgery [1,2].

## Case presentation

We present a 65-year-old white male who had been referred by his general practitioner for surgery because of worsening LUTS refractory to treatment with an alpha-blocker. Preoperatively, urine dipstick was clear, the prostate was measured by transrectal ultrasound at 55cc, International Prostate Symptom Score (IPSS) score was 20 and digital rectal examination showed a smooth, benign-feeling gland. Treatment was performed using the 180 W XPS GreenLight^TM^ laser (Boston Scientific, Marlborough, MA). The total operation time was 51 minutes, total lasering time was 32 minutes and 229 kJ of energy was delivered. The operative procedure was uncomplicated, and continuous bladder irrigation was stopped after three hours. The patient failed a trial without catheter the next day and following an uneventful recatheterization, he was discharged. 

A week after his discharge, he presented to the acute and emergency department with a left thigh pain transmitted to his left groin on exercise and mild LUTS. The district nurse removed his catheter the previous day as per local guidelines management and since then he had difficulties in walking despite adequate analgesic medication. On physical examination, his left proximal thigh was tender to palpation, oedematous and slightly erythymatous. He developed fever on the day of his readmission, and empirical antibiotic treatment with intravenous amoxicillin and clavulanic acid was commenced on the basis of previous sensitivity and local microbiology guidelines. Urinalysis in the emergency department demonstrated microscopic haematuria, leucocytes and nitrites, and urine culture revealed >108 cfu/L of ß-haemolytic Streptococcus group B susceptible to amoxycillin, trimethoprim and nitrofurantoin. His blood results demonstrated marked leucocytosis at 18x10^9^/L and elevated C-reactive protein.

**Figure 1:**
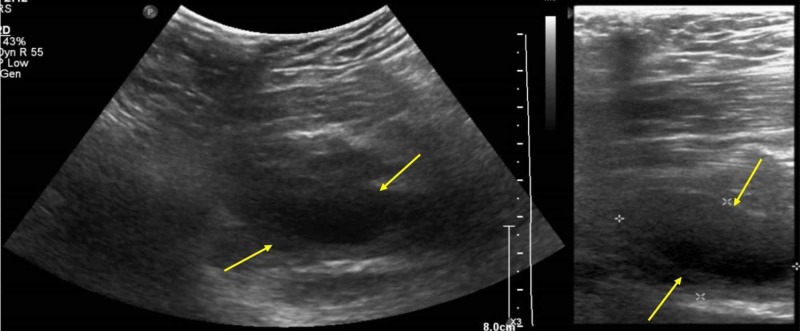
Soft tissue ultrasound scan: collection of fluid within the left adductor muscle and early cellulitis.

On the day of admission, he was subjected to an X-ray of his left thigh, which did not show any abnormality, and subsequently to an ultrasound (U/S) scan of the region which demonstrated a 14 x 4 cm collection within the left adductor muscle (Figure 1).

**Figure 2:**
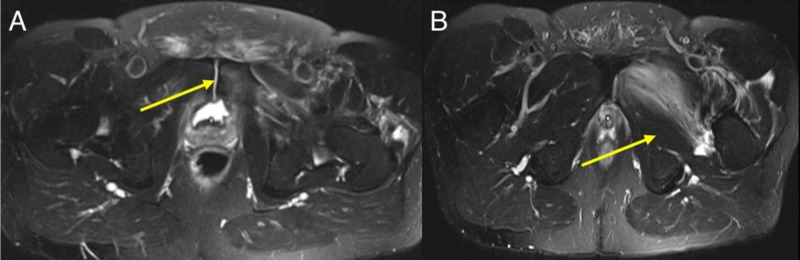
(A) MRI image shows fluid signal which appears to communicate from the bladder neck via the pubic symphysis inferolaterally into the left adductor collection with an adductor aponeurosis cleft sign noted. (B) Another MRI image shows a left-sided intramuscular collection with a maximally 6.5-cm transverse dimension and a 2.3-cm depth fluid collection noted centred upon the left adductor magnus muscle in the proximal thigh with marked surrounding oedema also involving the adductor brevis muscle.

On further MRI investigation the next day, a left-sided intramuscular collection was confirmed with a maximally 6.5-cm transverse dimension and a 2.3-cm depth fluid collection noted centred upon the left adductor magnus muscle in the proximal thigh with marked surrounding oedema also involving the adductor brevis muscle. There was also further oedema within the medial aspect of the left pubic tubercle consistent with osteitis pubis (Figure 2).

Eventually, the suspected anterior prostate capsule perforation was confirmed. Initial thoughts about decompressing the adductor collection via an U/S-guided drainage procedure were abandoned since the patient became apyrexial, his symptoms and blood results improved markedly and the collection started to decrease in size on further follow-up U/S scans. He remained inpatient for four days being discharged with a two-way 16Fr catheter and oral antibiotic cover for two months as per urine culture.

**Figure 3:**
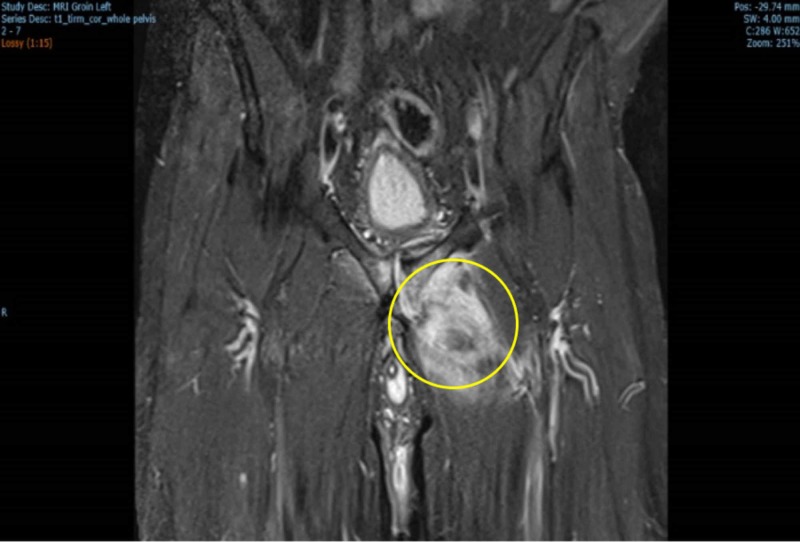
Coronal MRI image shows that the previously visualized fluid collection within the left adductor muscle compartment has partially resolved two months post-catherization. There remains however significant oedema within the muscle fibres. The ongoing communication from the bladder neck into the pubic symphysis is evident.

Two months after the operation, a repeat MRI of the pelvis showed only partial resolution of the urinoma (Figure 3) so we decided to leave the bladder catheter in place and refer the patient for a radical prostatectomy. An uneventful, nerve-sparing, robotic radical prostatectomy was performed one month later after which the patient is catheter free and continent.

## Discussion

The 180-W laser is more powerful than the preceding 80 W and 120 W lasers raising concerns regarding safety; however, the depth of tissue effect remains 1-2 mm suggesting this is a function of wavelength rather than power. PSFs are rare, and what makes them difficult to recognize is that patients often present with non-urological symptoms leading to a delay in diagnosis. The high index of suspicion on behalf of the surgeon is crucial for the initial diagnosis of this uncommon complication.

The aetiological background is hypothesized to be a capsular tear of the anterior prostatic tissue after prior transurethral resection of the prostate, PVP or radiotherapy. Few cases are reported in the literature after prostate cancer treatment, bladder tumor resection, transrectal biopsy of prostate or salvage cryotherapy [3]. Subsequent communication between the tissue planes might lead to anterior urinary extravasation, osteitis pubis and formation of urinoma. The spectrum of therapy extends from conservative treatment with long-term catheter and antibiotics to reconstruction with pubic symphysis debridement and fistula closure using an adjunct muscle, omental or peritoneal interposition flaps, radical prostatectomy and even urinary diversion [4,5].

## Conclusions

Our case highlights the challenge of PSF as rare clinical complication which is both difficult to diagnose and treat. Minimal use of high-power laser application in the anterior prostate tissue and high visibility of the appropriate surgical anatomical planes during the procedure are recommended. Regardless of initial conservative therapy, almost all patients will ultimately become recipients of a major invasive procedure.
